# Mucin-degrading gut commensals isolated from healthy faecal donor suppress intestinal epithelial inflammation and regulate tight junction barrier function

**DOI:** 10.3389/fimmu.2022.1021094

**Published:** 2022-10-12

**Authors:** Mingfang Pan, Nilakshi Barua, Margaret Ip

**Affiliations:** ^1^ Department of Microbiology, Faculty of Medicine, The Chinese University of Hong Kong, Shatin, Hong Kong SAR, China; ^2^ Centre for Gut Microbiota Research, Faculty of Medicine, The Chinese University of Hong Kong, Shatin, Hong Kong SAR, China

**Keywords:** mucin-degrading bacteria, 16S rRNA gene sequencing, tight junction barrier, gut commensal, Transepithelial Electrical Resistance (TEER), intestinal inflammation

## Abstract

The intestinal epithelium surface is covered by a layer of mucus that harbors a complex and dynamic population of bacteria termed gut microbiota. In particular, some gut bacteria have the ability to degrade the mucin glycan for nutritional sources. However, the bacterial diversity of mucin-degrading bacteria in human gut microbiota and their role in the gut remains unclear. In this study, we characterized the diversity of mucin-degrading bacteria in the human gut microbiota by an established cultivation-based molecular profiling method. The results showed the gut commensals having the mucin degrading ability were widely distributed in the gut microbiota and were more abundant than previously thought. In addition, many previously uncharacterized mucin degraders were isolated from faecals samples, suggesting the mucin-degrading gut commensals were underappreciated. To gain a better understanding of the interaction between these mucin-degrading gut commensals and the host, the effect of the commensals on intestinal epithelial cells were examined, and the results revealed that the commensals (8 *Bacteroides* spp., 2 *Parabacteroides* spp, *Akkermanisa muciniphila* and *Bifidobacterial dentium*) incited low level of inflammatory response (IL-8 and TNF-α) but suppressed the inflammatory response induced by *E. coli* through downregulating the NF-κB pathway. The presence of gut commensals also showed potential in enhancing the epithelial tight junction (TJ) barrier function through regulating the mRNA expression of TJ protein genes such as Zo-1, Occludin, Claudin-1 and E-cadherin. Furthermore, the presence of commensal bacteria *P. distasonis*, *B. thetaiotaomicron* and *A. muciniphila* completely or partly restored the pro-inflammatory cytokine IL-1β induced TJ barrier disruption. In conclusion, these findings indicate that mucin-degrading gut commensals were widely distributed in the gut microbiota and showed anti-inflammatory effect against pathogen infection and potential in modulating the epithelial barrier function.

## Introduction

The intestinal mucus layer acts as a line of defense that separates the luminal environment, including gut microbiota and other xenobiotics, from the host epithelial and immune cells (1). Intestinal mucin is mainly composed of a single highly O-glycosylated protein called mucin glycoproteins, with fucose or sialic acid terminating the glycan chains (2). Certain bacteria have developed various enzymatic machinery that can cleave and catabolize the sugar moieties for colonization. The fucosidase and sialidase activity of certain symbiotic bacteria, such as *Bacteroides thetaiotaomicron*, can liberate mucosal glycans without compromising the integrity of the mucus layer to support colonization (3). This ability helps the establishment of early colonization and offers ecological advantages to the gut commensals by providing an alternative endogenous source of nutrients when nutrition is depleted (4).

Once established in the intestine, the microbiota plays a vital role in human health and disease, influencing host immunity and metabolism. The impact is expected to be more direct for mucus-associated bacteria because of their proximity to the gut epithelial cells and immune system (3, 5). A typical example is *Akkermanisa muciniphila*, a recently isolated mucin degrader. Its abundance has been inversely correlated with the many diseases such as IBDs, type 1 diabetes mellitus, atopic dermatitis, autism, type 2 diabetes mellitus, and obesity (6). *A. muciniphila* has been proposed as a candidate next-generation probiotic because it has been demonstrated to possess many beneficial properties, including modulation of goblet cell number, mucus production, mucus barrier protection, and host immune modulation (7).

However, excess mucin degradation may disrupt the integrity of the mucosal layer and facilitate the access of luminal bacteria/antigens to the intestinal epithelial cells and immune system and then incite or exacerbate inflammation disease (8). Indeed, an increase in total mucosa-associated bacteria was observed in patients with inflammatory bowel disease (9). Furthermore, it has been reported that IBD patients have a disproportionate representation of mucin-degrading bacteria (10). A ∼100-fold and >4-fold increase in *Ruminococcus torques* and *R. gnavus*, respectively, was observed in macroscopically- and histologically-normal intestinal epithelia in cases of both Crohn’s disease (CD) and ulcerative colitis (UC). Together, these observations suggest the relevance of mucin degradation by the gut bacteria in human health and progress of the conditions such as gut inflammation disease. However, the systematic contribution of mucin-degraders in gut homeostasis and dysbiosis has not yet been thoroughly investigated, as only a limited number of mucin degraders and related enzymes have been studied (11).

In this study, we used a strategy combining both cultivation-dependent and -independent methods to characterize the diversity of human gut microbiota that can degrade mucin for growth. Concurrently, we isolated many potential, yet undescribed, mucin degraders from faecal samples. These mucin-degrading gut bacteria significantly suppressed the inflammatory response stimulated by *E. coli* and showed potential in modulating the epithelial tight junction (TJ) barrier function.

## Materials and methods

### Sample collection and process

Multiple faecal samples were collected from three healthy Hong Kong Chinese adults eligible for faeces donation for transplantation. A stringent set of criteria and screening as previously described was used to define its eligibility (12). Fresh faecal samples were kept on ice once being passed and were processed within two hours to preserve the viability of anaerobic bacteria as far as possible. Culture media and reagents for anaerobic cultures were placed in the anaerobic chamber (Bugbox Plus UM-017, Baker) overnight before use for pre-reduction of oxygen. The anaerobic chamber was held at 37 °C with 10% carbon dioxide, 10% hydrogen, 80% nitrogen. Weighted faecal sample (~ 1 g) was homogenized in reduced PBS by thorough vortex and then serially diluted tenfold down to 10^-2^. Plate culture was performed in the anaerobic chamber by spread plating 100 µl of the respective faecal dilutions on each petri dish. Mucin medium (MM) was prepared according to Wlodarska et al. (13) with some modifications and containing 0.5% porcine mucin (M1778, Sigma), 100 mM KH_2_PO_4_ (pH 7.2), 15 mM NaCl, 8.5 mM (NH4)_2_SO_4_, 4 mM L-cysteine, 11.1 mM vitamin K1, 15.3 mM hemin solution, 100 mM MgCl_2_, 1.4 mM FeSO_4_▪7H_2_O, 50 mM CaCl_2_, 1% trace mineral supplement (ATCC), and 1% vitamin supplement (ATCC). Plates were incubated anaerobically at 37 °C for 7 days. On day 7, plates containing confluent growth of discrete colonies were manually scraped off the media surface and rinsed with 5 ml PBS solution in 10 ml tubes. The collected tubes were centrifuged at 5, 000 g for 30 min at 4°C. The pelleted samples were stored at -80 °C for DNA extraction.

### Isolation and identification of potential mucin degraders

Fresh stool samples from the same subjects were used to make serial dilution and spread plate culture as described above. On day 7, colonies on the plates were picked and sub-cultured for identification by Matrix-Assisted Laser Desorption Ionization-Time of Flight (MALDI-TOF) mass spectrometry (Bruker Daltonics). For the isolates that identification scores below 1.7 were further identified by full-length 16S rRNA gene sequencing using one degenerate primer: 7F (5’-AGAGTTTGATYMTGGCTCAG-3’) forward primer and 1510R (5’-ACGGYTACCTTGTTACGACTT-3’) (14).

The mucin degrading ability of the isolates was tested by measuring the amount of mucin degraded in the supernatant using the anthrone reagent test as described before (10). In brief, after 72 h anaerobic culture in MM broth culture, an aliquot of 50 µL of the supernatants were loaded in 96-well microplate followed by 150 µL of anthrone reagent (0.1%). Subsequently, plates were incubated 20 min at 100°C in water bath followed by a cooling step treatment at room temperature before reading absorbance at 620 nm. Measurements were taken in triplicates. Colorimetric absorbance was compared to a standard curve based on glucose, and total carbohydrate content was expressed as μg/mL of glucose. Anthrone reagent tests were repeated three times.

### Faecal DNA extraction, 16S rRNA gene sequencing and analysis

Faecal DNA was extracted using the DNeasy Power soil kit (Qiagen). Following extraction, the V3-V4 variable regions of the 16S rRNA gene were amplified by polymerase chain reaction (PCR) using forward (341F: CCTACGGGNGGCWGCAG) and reverse (806R: GGACTACNVGGGTWTCTAAT) primers (15). A second PCR was applied to add the sequencing adapters and multiplex indices. PCR products were purified using the MEGAquick-spin™ Total Fragment DNA Purification Kit (iNtRON Biotechnology). 2 × 300 bp sequencing was performed on an Illumina MiSeq platform (outsourced to the Genomics Resource Core Facility at Cornell University, New York). Approximately 40,000 reads were sequenced per sample. QIIME 2 pipeline (v2019.1) was employed to process the raw sequencing files (https://qiime2.org/). Briefly, paired-end demultiplexed fastq sequence data were imported into QIIME and de-noised by DADA2 workflow (16). OTUs with a total frequency of less than 10 was filtered from the OTU table produced by DADA2 since the number of reads between replicates was less reproducible below this depth (17). For taxonomy classification, a pre-trained classifier based on Silva database (https://www.arb-silva.de/download/archive/qiime/) was used to assign taxonomy to the representative sequences. Phylogenetic trees and dendrograms were visualized using iTOL.

### Intestinal epithelial cell (IEC) culture

The colorectal adenocarcinoma cell line HT-29 cells and Caco-2 cells were maintained at 37°C in 5% CO_2_ and 95% air in Dulbecco’s Modified Eagle Medium (DMEM) and McCoy 5a media, respectively. 10% inactivated fetal calf serum (Gibco) and 1% penicillin-streptomycin (Sigma-Aldrich) were supplemented as necessary. The medium was changed every second day. At ~80% confluence, HT-29 cells were harvested by adding trypsin-EDTA solution (Invitrogen) (0.25% [wt/vol] trypsin–1 mM EDTA) and counted in a hemocytometer before seeding in 24-well tissue culture plates (Costar) at a concentration of 5×10^4^ cells/well. HT-29 cells were cultured until the complete confluence had been reached. Complete growth medium without the antibiotics was added, and the assay was performed the following day. Caco-2 cells were seeded on 12-well transwell inserts (12 mm diameter, 0.4 μm pore size, Polyester Membrane, Costar) at a concentration of 1×10^5^ cells/mL. The cells were cultured until the cell monolayer had been formed and differentiated, i.e., around 14-17 days post seeding according to previous publications (18, 19). At this stage, the transepithelial resistance between the apical and basolateral surfaces of the cell monolayers reaches a relatively stable status and is in the appropriate growth phase to evaluate how it is affected by external factors.

### Bacterial treatment

Prior to bacterial treatment, the inoculums of the commensals were centrifuged at 10,000 g for 10 min. The bacterial pellet was suspended in PBS and adjusted to McFarland 1.0 which equals a bacterial concentration of around 3×10^8^ CFU/mL. The bacterial suspension was centrifuged and then re-suspended in cell culture media for bacteria-cell co-culture with a multiplicity of infection (MOI) of 100.

### Prevention and competition assay

Prevention and competition assay procedures were employed to evaluate the protective effect of the gut commensals on the IEC against pathogenic *E. coli* NCTC9001 infection. For the competition assays, gut commensals and *E. coli* NCTC9001 were added to the HT-29 cells and incubated at 37 °C for 2 h. For the prevention assays, cells were preincubated with gut commensals at 37 °C for two hours. Following incubation, the unattached bacteria were removed by washing the monolayers with sterile PBS three times and then *E. coli* NCTC9001 was added. Cell monolayer co-culture with gut commensals or *E. coli* NCTC9001 was used as a control. Cells were then detached from the wells by incubating with 100 uL trypsin-EDTA per well for 5 min at 37°C. The bacteria attached to the cells were determined by the plating counting method after serial 10-fold dilutions. Adhesion results are presented as percentage of adhesion, as obtained by dividing the final count of attached bacteria by the initial bacterial number applied to co-culture.

### Cell RNA extraction and qRT-PCR

Total RNA from the cells was extracted using TRIzol reagent (Life Technologies, CA, USA) according to the manufacturer’s manual instructions. TURBO DNA-free™ Kit (Ambion, TX, USA) was used to remove genomic DNA in RNA before cDNA synthesis. The cDNA was synthesized using the SuperScript™ III First-Strand Synthesis System (Invitrogen, CA, USA) following the manufacturer’s instructions. TJ- and immune response-related genes were quantified using SYBR Green PCR Master Mix (Invitrogen, CA, USA). The primer sequences are listed in **Tables S1**, **S2**. The amplification program consisted of a pre-cycling hold at 95°C for 10 min, followed by 40 cycles of denaturation at 95°C for 15 s, annealing at 60°C for 30 s, and extension at 60°C for 30 s. Applied Biosystems 7500 Real-Time PCR System (Applied Biosystems, MA, USA) was used to analyze the mRNA expression levels. The gene’s mRNA expression was normalized to the levels of 18s rRNA and expressed as the fold change relative to untreated cells using the 2^–ΔΔCt^ method. Reactions were performed in at least three replicates.

### Measurement of Transepithelial Electrical Resistance

Transepithelial Electrical Resistance (TEER) is an indicator of epithelial paracellular permeability to ionic solutes and was used to assess intestinal barrier function. The TEER values were measured using a Millicell Electrical Resistance System (ERS-2) meter (Millipore Corporation, Bedford, MA, USA). The TEER value of the cell monolayer is calculated by the equation: TEER = (Rm − Ri) × A. Rm is the transmembrane resistance of the treated group, Ri is the intrinsic resistance of a cell-free transwell, and A is the surface area (cm^2^) of the membrane of the insert. The protective effect of gut isolates on *E. coli*-induced intestinal permeability dysfunction was measured as follows: differentiated Caco-2 cell monolayers were treated with pathogenic *E. coli* together with gut isolates, the mixture was incubated for 2 h. The TEER value was measured before the addition of the bacteria (time zero) and then at various time intervals and expressed as the ratio of TEER at time ‘*t’* in relation to the initial value (at time zero) for each series. In parallel, cell monolayers mono-cultured with the respective strains represented the control for each experiment.

### Immunofluorescence staining

After bacteria-cell co-culture, Caco-2 cell monolayers were washed with PBS before being fixed with 100% ice-cold methanal at -20 °C for 5 min and permeabilized with 0.5% Triton X-100 in PBS room temperature for 10 min. The monolayers were then washed with PBS and blocked for 1 hour with 4% (w/v) bovine serum albumin (BSA) at room temperature. The cells were then incubated with either antibody of Zo-1(Cell Signaling Technology,13663S), Occludin (Cell Signaling Technology, 91131S), or E-cadherin (Cell Signaling Technology, 3195S). Cells were fixed with 4% paraformaldehyde when staining the Claudin-2 with Claudin-2 (Invitrogen, 51–6100) primary antibodies. The cells were rinsed again with washing PBS three times, followed by incubation with the Alexa Fluor 594 goat anti-rabbit secondary antibody (Cell Signaling Technology, 8889S) for 1 h at room temperature. The cells were rinsed again with PBS before the membranes were separated from the Transwell insert using a scalpel. The membrane was finally mounted with cell side up between a slide and coverslip with UltraCruz^®^ Aqueous Mounting Medium (Santa Cruz Biotechnology). The mounting media contains DAPI to stain all the nuclei. The microscopy of the mounted membranes was performed on a Nikon Confocal Laser Scanning Microscope.

### Data availability

All 16S rRNA sequence data generated from this study were deposited to the GeneBank Sequence Read Archive with accession number PRJNA635743.

### Statistical analysis

All statistical analyses were performed using GraphPad Prism 9 (GraphPad Software, Inc.). Statistical differences between experimental groups were evaluated by Student’s t tests and one-way analysis of variance (ANOVA) with a Dunnett’s test for multiple comparisons. All data were expressed as means ± standard deviations (SD). *p* values of < 0.05 were considered statistically significant.

## Results

### Targeted culture-dependent molecular profiling identifies mucin-degrading bacteria in the healthy intestinal microbiome

**Figure 1:**
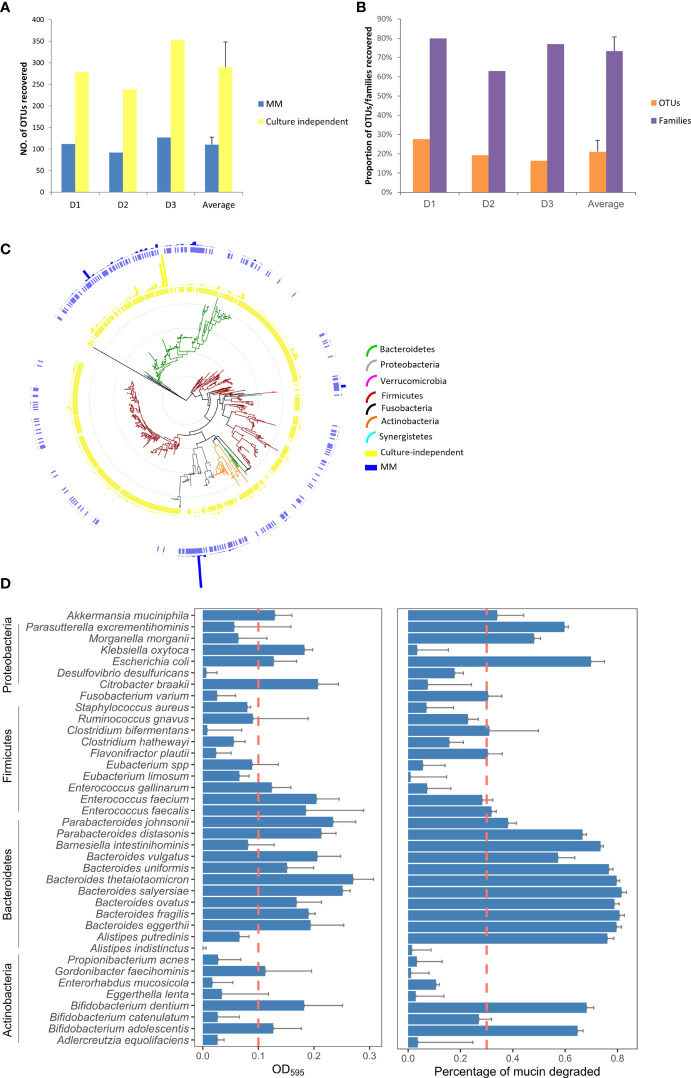
Mucin-degrading bacteria captured by cultivation-based molecular profiling. **(A)** Number of OTUs recovered from 16S rRNA gene sequencing of faecal samples from the three health Donor 1 (D1), Donor 2 (D2) and Donor 3 (D3) by MM media and culture-independent methods. **(B)** Proportion of OTUs and the corresponding bacterial families from culture MM media as compared to the culture-independent sequencing method. **(C)** The phylogenetic tree and relative abundance of the OTUs recovered from 16S rRNA gene sequencing of the faecal samples by MM culture and culture-independent methods. The inner ring is colored based on the phylum the OTUs are assigned. The outer ring shows the distribution of OTUs. The exterior bar chart shows the relative abundance of the OTUs. **(D)** Percentage of mucin degraded over 72 h by the 38 gut isolates in minimal media containing porcine gastric mucin as the sole carbon source. The amount of mucin degraded was measured by the loss of hexose in the supernatant using the anthrone reagent test. Data are means and standard deviations for three replicates.

A targeted culture-dependent molecular profiling method was developed to gain a whole picture of the bacterial diversity of mucin-degrading bacteria in the human gut microbiota. A minimal culture media with mucin as the sole carbon source (MM media) was used to enrich mucin-degrading bacteria from faecal samples selectively. Then the colonies grow on the plates was applied to 16S rRNA gene sequencing for bacterial diversity analysis. Faecal samples without culture (culture-independent) were used as control. On average, 110 ± 18 OTUs per subject were recovered by MM media. Meanwhile, 290 ± 58 OTUs per subject were recovered by cultured-independent sequencing method (**Figure 1A**). In comparison, 21.1% of the OTUs detected from faecal samples by culture-independent sequencing were cultured by MM media, representing 73.3% of family-level taxonomic groups from the faecal samples (**Figure 1B**). Taxonomic analysis showed the OTUs recovered from MM media (**Figure 1C**, outer ring in blue) were widely distributed in the seven phyla, namely Firmicutes, Bacteroidetes, Proteobacteria, Verrucomicrobia, Actinobacteria, Fusobacteria, and Synergistetes (**Figure 1C**, inner tree). A large proportion of the OTUs enriched by MM belong to the phylum of Proteobacteria (40.4%) and Bacteroidetes (49.4%), dominated by the bacterial species including *Escherichia* (37.8%), *Parabacteroides distasonis* (16.1%), *Bacteroides fragilis* (14.0%), *Bacteroides vulgatus* (8.6%), *Bacteroides dorei* (2.8%), *Bacteroides thetaiotaomicron* (1.4%) and other *Bacteroides species* (**Figure 1C**, the outer blue bar chart). Notably, 8.6% of OTUs falls under the phylum of Verrucomicrobia (*Akkermanisa*). MM media also enriched the broad bacterial diversity of OTUs belonging to Firmicutes. However, the relative abundance was relatively low (1.2%), indicating that most MM-enriched OTUs from the Firmicutes were rare bacterial species/strains. In addition, 0.32% and 0.01% of OTUs belonging to the phyla of Fusobacteria (*Fusobacterium*) and Synergistetes (*Cloacibacillus*), respectively were detected in MM as well (**Figure 1C**, the outer blue bar chart).

To further test the distribution of mucin degradation in the human gut microbiota, we screen 38 bacterial species for growth in minimal media broth containing commercially available pig-derived gastric mucin as a sole carbon source. These 38 bacterial species belong to 24 genera and represent 70.4% of the bacterial abundance at the genus level in faecal samples as determined by 16S rRNA gene sequence (**Figure S1**). 50% (19 out of the 38) test bacterial species are able to grow in MM media (change of OD_595_ >0.1) (**Figure 1D**). Consistent with the bacterial growth, most of them showed > 30% reduction of mucin in the broth as determined by the anthrone reagent test (**Figure 1D**), suggesting that the mucus-utilizing capacity of the intestinal microbiome has been underappreciated. Apart from the known mucin degraders like *A. muciniphila*, *B. thetaiotaomicron*, *B. fragilis* and *B. vulgatus*, importantly, we found many other bacterial species showed potential mucin-degrading ability as well. This potential, yet undescribed, mucin degraders include five *Bacteroides* (*B. intestinihominis, B. uniformis, B. salyersiae, B. ovatus* and *B. eggerthii*) and two *Parabacteroides* strains (*P. johnsonii* and *P. distasonis*), two *Bifidobacterium* strains (*B. dentium* and *B. adolescentis*), *Parasutterella excrementihominis*, *Gordonibacter faecihominis* and *Klebsiella oxytoca.*

### Intestinal epithelial cells immune tolerance to the mucin-degrading gut commensals

**Figure 2:**
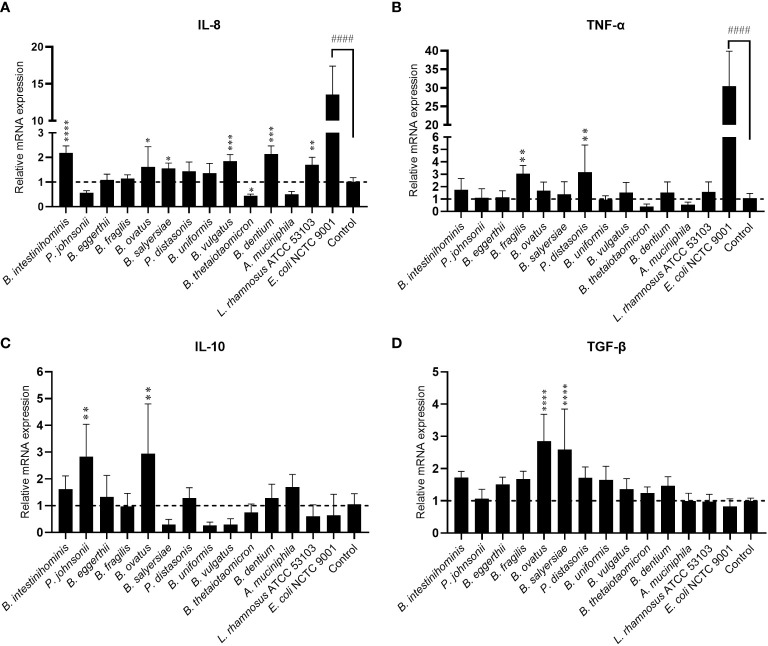
Effects of mucin-degrading gut commensals on the mRNA expression of IL-8 **(A)**, TNF-α **(B)**, IL-10 **(C)** and TGF-β **(D)** in HT-29 after 2-hour co-culture. *L. rhamnosus* ATCC 53103 and pathogenic *E. coli* NCTC9001 were included in the experiments as control strains. ####*p* < 0.0001 compared to the control group; *p < 0.05, **p < 0.01, ***p < 0.001, ****p < 0.0001 compared to the *E. coli* NCTC9001 group as determined by one-way ANOVA followed by Dunnett’s test for multiple comparisons.

Gut commensals with mucin-degrading ability are expected to have more chance to interact with mucosal immunity. We then evaluated the immunomodulating effect of 12 selected gut commensals with relatively high mucin-degrading ability by co-culture with the intestinal epithelial cells line HT-29. Real-time PCR was conducted to measure the transcription of pro-inflammatory cytokines IL-8 and TNF-α, and anti-inflammatory cytokines IL-10 and TGF-β. As a positive control, *E. coli* NCTC 9001 stimulated high-level mRNA expression of pro-inflammatory cytokines IL-8 (13.6 ± 3.9 fold) (**Figure 2A**) and TNF-α (30.5 ± 9.4 fold) (**Figure 2B**) but not anti-inflammatory cytokines IL-10 (0.64 ± 0.78 fold) (**Figure 2C** **)** and TGF-β (0.83 ± 0.24 fold) (**Figure 2D** **)**. In contrast, most isolated mucin-degrading gut commensals induce small or little mRNA expression of pro-inflammatory cytokines (**Figure 2**). Four *Bacteroides* and *B. dentium* induced ~2-fold increase of IL-8 (**Figure 2A**), while two *Bacteroides* stimulated around 3-fold increase of TNF-α (**Figure 2B**). Interestingly, *P. johnsonii*, *B. thetaiotaomicron* and *A. muciniphila* were able to down-regulate the cell basal level of IL-8 (~0.5 fold) or TNF-α (~0.5 fold) (**Figures 2A, B**). In addition, *P. johnsonii*, *B. ovatus* and *B. salyersiae* were able to induce anti-inflammatory cytokines IL-10 or TGF-β (**Figures 2C, D**). These observations suggested the immune tolerance of these mucin-degrading gut commensals to intestinal epithelial cells.

### Mucin-utilizing gut commensal alleviate inflammation induced by pathogenic *E. coli*

**Figure 3:**
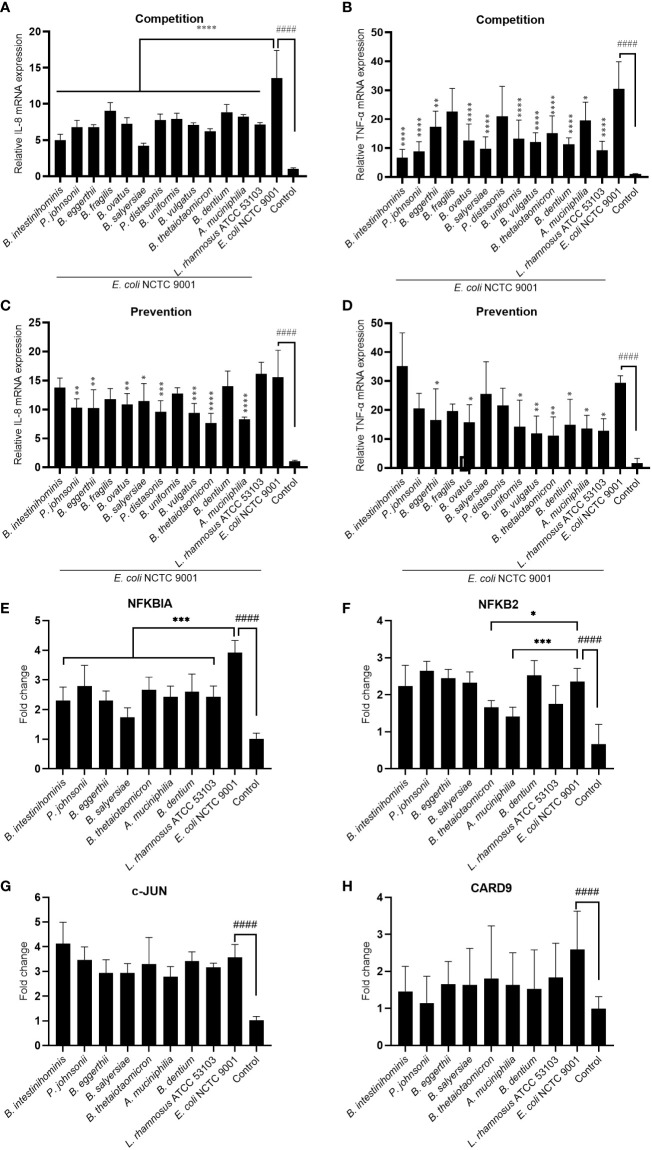
Mucin-degrading gut commensals alleviate the pathogenic *E. coli* induced inflammation by downregulating the NF-kB signaling pathway. HT-29 cells co-incubated (competition) or pre-incubated (prevention) with gut commensals were challenged by *E. coli* 2 h. The IL-8 **(A, C)** and TNF-α **(B, D)** mRNA expression in HT-29 cells was detected by qPCR. HT-29 cells incubated with gut commensals were challenged by *E. coli* NCTC9001 for 2 h, mRNA expression of NF-kB and MAPK pathway involved gene NFKBIA **(E)**, NFKB2 **(F)**, c-Jun **(G)** and CARD 9 **(H)** were measured by qPCR. All data are presented as the mean ± SD of three biological replicates with two technical replicates. ####p < 0.0001 compared to the control group; *p < 0.05, **p < 0.01, ***p < 0.001, ****p < 0.0001 compared to the *E. coli* NCTC9001 group as determined by one-way ANOVA followed by Dunnett**’**s test for multiple comparisons.

To test the hypothesis that these mucin-degrading commensals have an anti-inflammation function in the intestine, we treated the HT-29 cell with selected mucin-degrading gut commensals followed by stimulation with *E. coli* NCTC9001 and examined the mRNA expression of pro-inflammatory cytokines IL-8 and TNF-α. As *L. rhamnosus* is known to have anti-inflammatory potential, we used this strain as a positive control. As shown in **Figures 3A, B**, the presence of all the isolated gut commensals, as well as the *L. rhamnosus* ATCC 53103, significantly decreased the *E. coli* induced IL-8 and TNF-α, except for *B. fragilis* and *P. distasonis* which showed no impact on the downregulation of *E. coli* induced mRNA expression of TNF-α. This suggests that anti-inflammation seems a common but bacterial species-specific property of the mucin-degrading gut commensals. Compared to the competition co-culture model, however, pretreatment of the cells with the gut commensals showed compromised effects on down-regulating the IL-8 and TNF-α (**Figures 3C, D**). For example, *B. intestinihominis* and *P. johnsonii*, were the best in decreasing the *E. coli*-induced TNF-α in the competition assay but showed no significant effect on the TNF-α level in the pretreatment assay. This result suggests that efficient anti-inflammation requires the presence of gut commensals.

Antagonization of the adhesion of pathogen to the epithelial cell is one of the mechanism that mediated the anti-inflammatory effect of some probiotics. However, no significant antagonization of the *E. coli* NCTC9001 adhesion to HT-29 cells was observed in either prevention (**Figure S2A**) or competition model (**Figure S2B**). This data suggests the anti-inflammation property of these gut commensals observed above is not attributed to the antagonism of pathogen adhesion to HT-29 cells but may be due to direct bacteria-cell interaction. We further explored two intracellular signaling pathways involved in the regulation of immune response: NF-kB and MAPK pathways. *E. coli* induced an activation of both NF-kB and MAPK pathways (**Figure 3** and **Figure S3**) as shown by a significant increase in NFKBIA (**Figure 3E**), NFKB2 (**Figure 3F**), c-Jun (**Figure 3G**) and CARD9 (H). However, all the selected gut commensals, together with the control *L. rhamnosus* ATCC 53103, significantly downregulated the *E. coli*-induced NFKBIA (**Figure 3E**). In addition, *B. thetaiotaomicron* and *A. muciniphila* significantly suppressed the *E. coli* induced NFKB2 (**Figure 3F**). However, these gut commensals have no effect on the *E. coli*-induced c-Jun (**Figure 3G**) and CARD9 (H), suggesting the anti-inflammation exert by gut commensals is due to the inhibition of NF-kB signaling pathway but not the MAPK pathway.

### Mucin-degrading gut commensals affect Caco-2 intestinal epithelial TJ barrier function and mRNA expression of TJ protein genes

**Figure 4:**
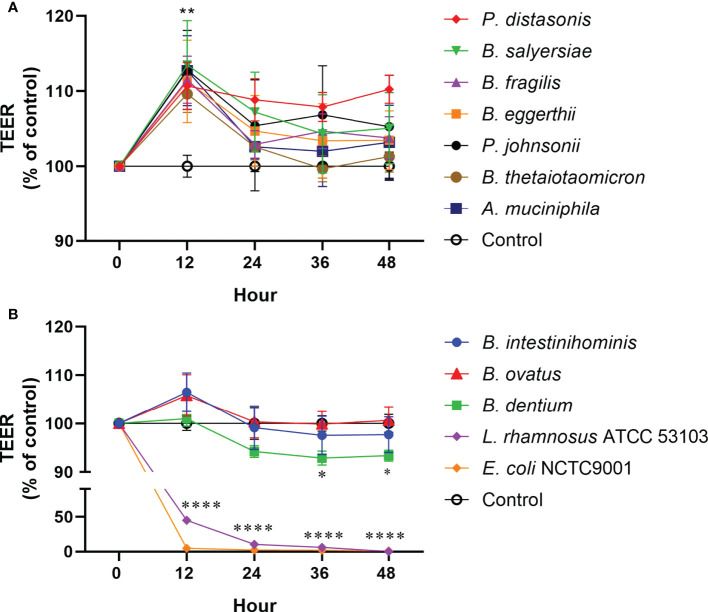
Effect of mucin-degrading gut commensals on the development of the TEER of Caco-2 monolayer. Changes in the TEER values of the Caco-2 cell monolayers co-cultured with bacteria were monitored over 48** **h experiment. Cell monolayers without bacteria incubation were used as the control group and the TEER values were normalized to 100%. *p < 0.05, **p < 0.01, ****p < 0.0001 treated groups vs. control group. Error bars represent the standard deviation of the mean values from at least three replicates. Significance was determined by one-way ANOVA analysis.

To examine the effect of mucin-degrading gut commensals on the epithelial TJ barrier function, the influence of 10 selected mucin-degrading gut commensals (2 *Parabacteroides* spp., 6 *Bacteroides* spp., 1 *B. dentium* and 1 A*. muciniphilia*) on TEER value of filter grown Caco-2 cell monolayers was tested. Cell monolayers without bacterial incubation were considered as a control group. Changes in the TEER value after co-culture were used as a readout parameter for potential effects on intestinal epithelial TJ barrier function. As shown in **Figure 4A**, 2 *Parabacteroides* spp., 4 *Bacteroides* spp., and *A. muciniphilia* significantly increased the TEER value of Caco-2 cell monolayers by around 10% after 12-hour coincubation. However, two *Bacteroide* species (*B. intestinihominis* and *B. ovatus*) had no significant effects on the TEER value over the 48 hours of co-incubation (**Figure 4B**). In contrast, *B. dentium* and the control strain *L. rhamnosus* ATCC 53103 and *E. coli* NCTC 9001 significantly decreased the TEER value after co-incubation (**Figure 4B**). Together, these results suggest most of the test mucin-degrading gut commensals could enhance intestinal epitheal TJ barrier function.

**Figure 5:**
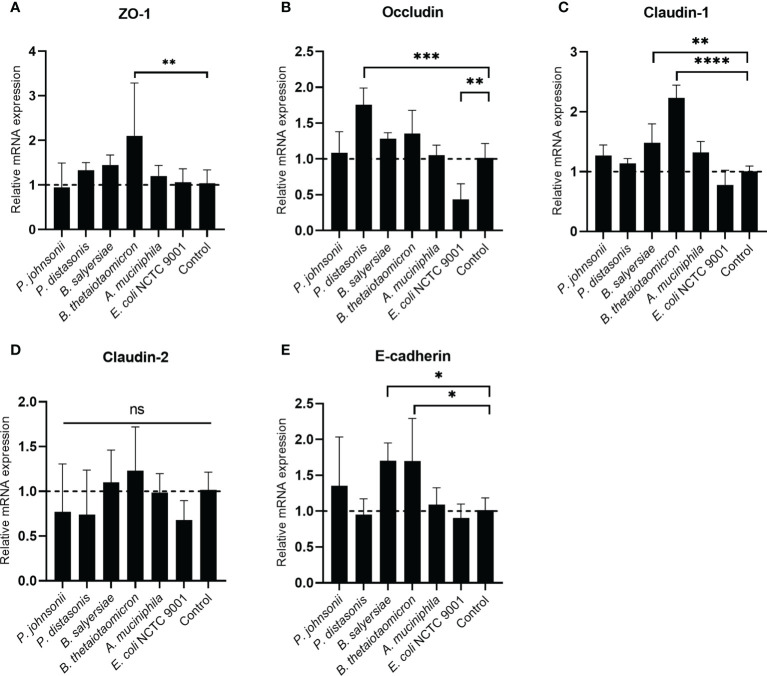
Impact of selected mucin-degrading gut commensals on the mRNA expression of TJ protein genes. Developed Caco-2 cell monolayers were co-cultured with gut commensals 24 h. The mRNA level of TJ protein Zo-1 **(A)**, Occludin **(B)** Claudin-1 **(C)**, E-cadherin **(D)** and Claduin-2 **(E)** were detected by qRT-PCR. Error bars represent the standard deviation of the mean values from at least three replicates. *p < 0.05, **p < 0.01, ***p < 0.001, ****p < 0.0001 treated groups vs. control group, ns: no significant. Significance was determined by one-way ANOVA followed by Dunnett**’**s test for multiple comparisons.

The TJ proteins are the primary determinants of intestinal epithelial TJ barrier function (20). Therefore, we examined if the regulation of TJ proteins mediated the enhancement of the epithelial barrier function by the gut commensals. The transcription of TJ protein genes, including Zo-1, Occludin, Claudin-1, Caludin-2 and E-cadherin, was determined by measuring the mRNA level through qRT-PCR (**Figure 5**). we found the effect of mucin-degrading gut commensals on the mRNA expression of TJ protein genes is bacteria-specific. After co-incubation with Caco-2 cell monolayer, *B. thetaiotaomicron* significantly upregulated the mRNA level of Zo-1 (**Figure 5A**), Claudin-1(**Figure 5C**) and E-cadherin (**Figure 5E**). At the same time, *P. distasonis* significantly increased the Occludin (**Figure 5B**) while *B. salyersiae* significantly upregulated Claudin-1 (**Figure 5C**) and E-cadherin (**Figure 5E**). In contrast, the control strain *E. coli* NCTC 9001 significantly downregulated the Occludin mRNA level (**Figure 5B**). *P. johnsonii* had on significant impact on the mRNA expression of TJ protein genes. In addition, all the selected gut commensals and *E. coli* NCTC 9001 did not affect the expression of Claudin-2 mRNA (**Figure 5D**).

### Protective effect of gut commensals on the intestinal barrier challenged by an inflammatory stimulus

**Figure 6:**
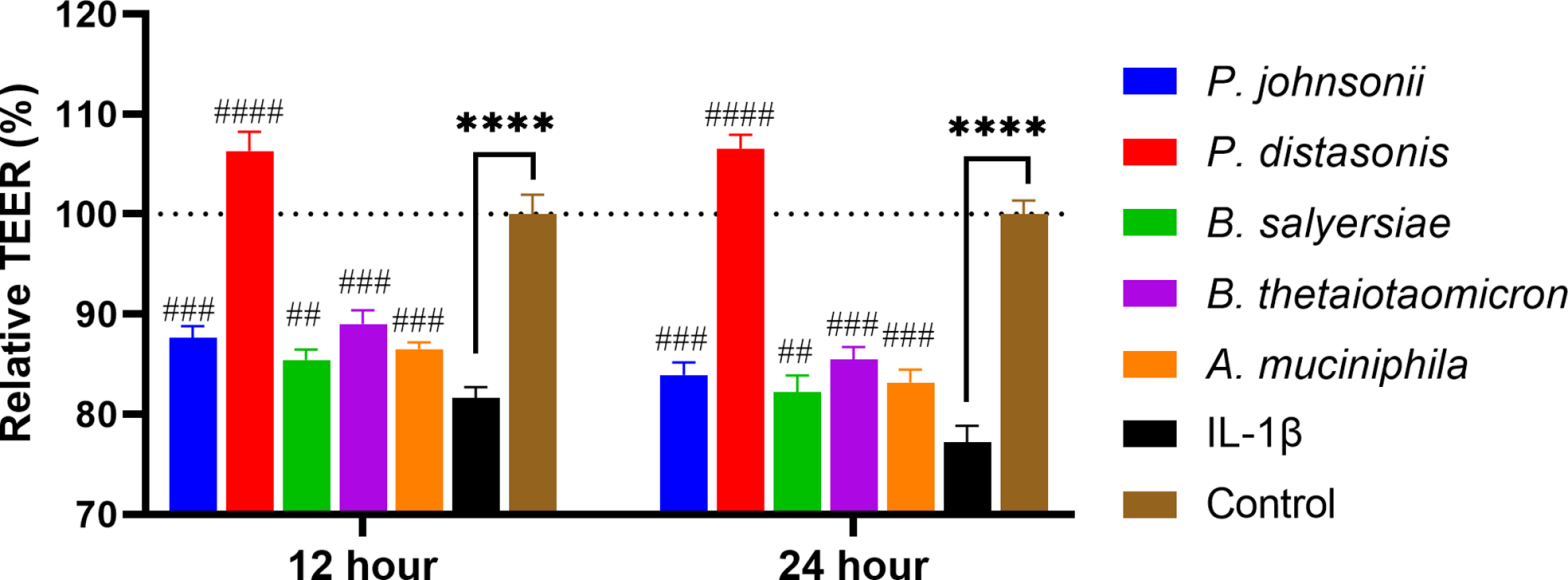
Selected mucin-degrading gut commensals attenuated the drop of TEER in Caco-2 cell monolayers induced by IL-1β. Caco-2 cell monolayers treated with gut commensals were followed by stimulation by IL-1β (10 ng/mL). The TEER value was measured at 12 h and 24 h time points. ****p < 0.0001 IL-1β vs. control. ##p < 0.01, ###p < 0.001, ####p < 0.0001 IL-1β vs. treated groups. Cells without any treatment were used as a control. Error bars represent the standard deviation of the mean values from at least three replicates. Significance was determined by one-way ANOVA followed by Dunnett**’**s test for multiple comparisons.

Inflammatory cytokines like IL-1β have been demonstrated to cause inflammation in intestinal epithelial cells and compromise intestinal epithelial TJ barrier function (21). We hypothesized that the selected gut commensals that exhibited an enhanced barrier function as shown above could protect the intestinal epithelial cell barrier against disruption induced by IL-1β. As shown in **Figure 6**, the addition of IL-1β at physiological concentration (10 ng/mL) to Caco-2 cell monolayers caused a significant and time-dependent drop in the TEER value, decreasing to 81.7% after 12 hours and 77.2% after 24 h when compared to control (100%). The presence of *P. distasonis* prevented the decrease of TEER induced by IL-1β, with the corresponding relative TEER value increased to ~106%. However, the other gut commensals, including *P. johnsonii*, *P. distasonis*, *B. salyersiae*, *B. thetaiotaomicron* and *A. muciniphila* only partially attenuated the decrease of TEER value induced by IL-1β.

### Mucin-degrading gut commensals restore IL-1 β induced dysregulation of TJ proteins and immune response

**Figure 7:**
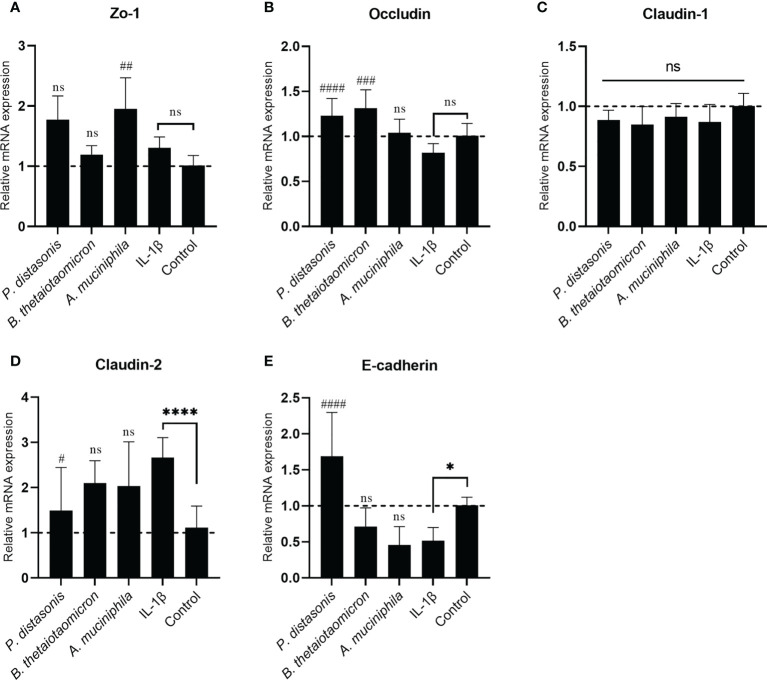
Co-culture with *P. distasonis, B. thetaiotaomicron* and *A*. *muciniphila* restored the IL-1β induced dysregulation of intestinal TJ protein genes’ mRNA expression. Caco-2 cell monolayers co-incubated with gut commensals were followed by stimulation by IL-1β (10 ng/mL). Cells without any treatment were used as a control. The mRNA expression of TJ protein Zo-1 **(A)**, Occludin **(B)**, Claudin-1 **(C)**, Claudin-2 **(D)** and E-cadherin **(E)** were measured by qPCR. Cells without any treatment were used as a control. *p < 0.05, ****p < 0.0001 IL-1β vs. control, ns: no significant. #p < 0.05, ##p < 0.01, ###p < 0.001, ####p < 0.0001 IL-1β vs. treated groups. Error bars represent the standard deviation of the mean values from at least three replicates. Significance was determined by one-way ANOVA followed by Dunnett**’**s test for multiple comparisons.

To investigate whether the changes in transepithelial resistance on epithelial cell monolayers following IL-1β and gut commensals treatments were associated with regulation of TJ proteins, the mRNA expression of the TJ protein genes Zo-1, Occludin, Claudin-1, Claudin-2 and E-cadherin were quantified by qRT-PCR analysis. The stimulation of Caco-2 cell monolayers with inflammatory cytokines IL-1 β did not affect the mRNA expression Zo-1(**Figure 7A**), Occludin (**Figure 7B**) and Claudin-1(**Figure 7C**), however, it caused a significant increase in Claudin-2 (2.66 ± 0.44 fold, *p*<0.01) (**Figure 7D**) and a significant decrease in E-cadherin (0.51 ± 0.18 fold, *p*<0.05) (**Figure 7E**). In addition, there was a small decrease trend in the Occludin (0.82 ± 0.10 fold, *p*>0.05) (**Figure 7B** **)**, but it did not reach statistical significance. After co-incubation with *P. distasonis* and *B. thetaiotaomicron* for 24 hours, a significantly increased in Occludin was observed compared to IL-1β group (**Figure 7B**). Furthermore, *P. distasonis* ameliorated the IL-1β-induced increase in Claudin-2 (**Figure 7D**) and decrease in E-cadherin (**Figure 7E**). In addition, we observed co-incubation with *A. muciniphila* significantly increased the mRNA level of Zo-1 (**Figure 7A**), although IL-1β did not affect Zo-1. Neither gut commensals nor IL-1β treatment caused a significant effect on the mRNA expression of Claudin-1 (**Figure 7C**).

Concurrently, the effect of these three gut commensals on the immune response was monitored as well. IL-1β (10 ng/mL) significantly increased the mRNA expression of NFKBIA gene (**Figure S4A**) and NF-kB downstream pro-inflammatory cytokine gene IL-8 (**Figure S4B**). The presence of *P. distasonis* and *A. muciniphila* significantly inhibited the IL-1β induced increase of NFKBIA and IL-8, suggesting a inhibition of NF-κB pathway by these two gut commensals. However, co-culture with *B. thetaiotaomicron* did not cause significant change in NFKBIA and IL-8.

### Gut commensals restore IL-1 β induced TJ protein localization disruption

**Figure 8:**
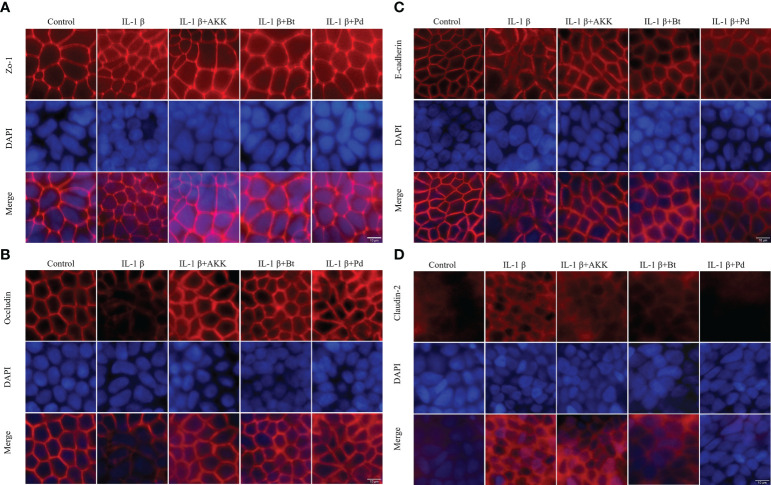
Protective effect of *P. distasonis* (Pd)*, A*. *muciniphila* (AKK) and *B. thetaiotaomicron* (Bt) on the immunofluorescence localization of TJ proteins in Caco-2 cell monolayers challenged by IL-1β. Caco-2 cell monolayers were treated with IL-1β and co-incubated with gut commensals for 24 h. TJ protein Zo-1 **(A)**, Occludin **(B)**, E-cadherin **(C)**, and Claudin-2 **(D)** were stained with the respective antibodies (red), and nuclei were DAPI stained (blue) and imaged by confocal microscopy. Images are of 100 × magnification. Scale Bar = 10 μm.

The expression and localization of the TJ proteins Zo-1, Occludin, Claudin-2, and E-cadherin were assessed by immunofluorescence staining. In control Caco-2 monolayers, Zo-1, Occludin, and E-cadherin staining formed a honeycomb-type structure outlining the cellular junctions (**Figure 8**). IL-1β did not cause an obvious change on Zo-1(**Figure 8A**). However, it caused a disturbance in the junctional localization of Occludin (**Figure 8B**) and E-cadherin (**Figure 8C** **)**. The faint immunofluorescence signal of Occludin staining at cell junctions indicated the synthesis of TJ protein was affected, and the irregular and distorted junctional location of E-cadherin suggested the TJ disruption. Co-incubation with *A. muciniphila*, *B. thetaiotaomicron* and *P. distasonis* restored the Occludin and E-cadherin at junctional localization areas (**Figures 8A, B**).

Moreover, Claudin-2 detection in control Caco-2 cell monolayers (**Figure 8D**) was low. However, an increased Claudin 2 signals was observed after IL-1β stimulation. The presence of *P. distasonis* remarkably prevented the IL-1β stimulated increase in the expression of Claudin-2 (**Figure 8D**). These results indicated that the intestinal epithelial TJ proteins were impaired after by the inflammatory cytokines, and supplementation of *P. distasonis, A. muciniphila* and *B. thetaiotaomicron* could completely or partly restore it.

## Discussion

Mucin degradation by the gut microbiota plays an essential role in the dynamic and homeostasis of mucus barrier. However, few publications well defined the proportion of this bacterial population with gut microbiota. Hoskins and Boulding (1981) estimated that an average 1% of the total faecal bacteria in healthy subjects were able to degrade the mucin glycoproteins based on the “most probable number (MPN)” method. However, *in silico* analysis of the genomes of gut microbiota revealed that up to 86% of the human gut microbiota encode genes for cleavage of mucin glycans and 89% encoding genes for the metabolism of the released monosaccharides, suggesting that the mucin-degrading bacteria in the human gut microbiota may have been underappreciated (22). In this study, through a targeted culture-based 16S rRNA gene sequencing, we showed 21.1% of the OTUs, representing 73.3% of family-level taxonomic groups, from faecal samples were able to grow on the MM media with porcine mucin as the sole carbon sources. *In vitro* monoculture screening of 38 bacterial species also found many potential, previously uncharacterized mucin degraders. Together, these results demonstrated that the mucin-degrading bacteria in the gut microbiota is more abundant than previously thought.

Mucin-degrading gut commensals are expected to interact intimately with the host epithelial immune system due to their close proximity to the gut epithelium and immune cells (23). However, the bacteria-cell co-culture results showed these isolated gut commensals (8 *Bacteroides* spp., 2 *Parabacteroides spp*, *Akkermanisa muciniphila* and *Bifidobacterial dentium*) elicit small amount mRNA expression of inflammatory cytokines IL-8 and TNF-α, suggesting the mucin-degrading gut commensals were tolerated by the host epithelial cells. Low or non-immunogenicity is important for gut commensals to escape the clearance by host immune response and reside in the GI tract (24). This is especially important in consideration of the mucus-associated gut commensals are in close proximity to host IEC.

Apart from low immunogenicity, we found all the selected mucin-degrading gut commensals could supress the IL-8 and TNF-α stimulated by *E. coli* at various degree, suggesting a potential of anti-inflammatory effects of these gut commensals. In recent years, a number of studies reported the anti-inflammatory property of bacteria and their potential application in the treatment of gut inflammation diseases like IBD (25). However, most of the related studies focus on probiotics like *Lactobacillus* and *Bifidobacterium* (26) and specific individual gut commensals like *Faecalibacterium prausnitzii* (27). The present study first described the anti-inflammatory property that seems common among mucin-degrading gut commensals, although this study does not explore whether there is a connection between anti-inflammatory property and mucin-degrading ability. In addition, we found the anti-inflammatory effect was more evident at the competition model than in prevention condition, suggesting the anti-inflammation needs the presence of gut commensals. Probiotics have been reported to exert the anti-inflammation effect by interference with pathogen binding to IEC (28). Therefore, we postulated that the anti-inflammatory property of the gut commensals tested above may be attributed to the same mechanism. However, the result revealed that mucin-degrading gut commensals have no significant antagonizing effect on *E. coli* adhesion to HT-29 cells, indicating the anti-inflammation property of the mucin-degrading gut commensals tested here is through other mechanisms. Further study of two intracellular signaling pathways, NF-kB pathway and MAPK pathway showed the isolated mucin-degrading gut commensals could significantly suppressed the NF-kB pathway by downregulation of the *E. coli* induced NFKBIA. However, no inhibitory effect on the MAPK pathway was observed. These results suggest that the commensals exert the anti-inflammatory effect by suppressing the NF-kB pathway but not the MAPK pathway. It also explains why the gut commensals inhibited only part of induced pro-inflammatory cytokines.

We further investigated the impact of these mucin-degrading bacteria on the intestinal epithelial barrier through a Caco-2 cell model. We thought this study is essential as mucin degradation by bacteria is considered a pathogenicity factor as excess mucin degradation disrupts the mucosal barrier (29). Impaired or compromised intestinal barrier function allows bacteria-derived molecules such as Lipopolysaccharides (LPS) into the mucosa and then cause intestinal and even systematic inflammatory responses (1). However, the results showed seven of the tested mucin-degrading gut commensals (2 *Parabacteroides* spp., 4 *Bacteroides* spp. and *A. muciniphilia*) significantly increased the TEER value of Caco-2 cell monolayers by around 10% after 12-hour co-incubation, suggesting an enhancement of the barrier function of the epithelial monolayer. The beneficial effect of probiotics such as *Lactobacillus* and *Bifidobacterium* on the intestinal epithelial barrier function have been investigated widely (30). Although the exact mechanism by which the bacteria exert this beneficial effect is not well defined, publications reveal that certain probiotic (e.g *Bifidobacterium* and *Lactobacillus* spp.) promote epithelial barrier function through regulation of TJ proteins (31, 32). To test if the mucin-degrading gut commensals isolated in this study act as the exact mechanism, mRNA expression of TJ protein genes was measured after co-culture. Indeed, the mRNA expression of TJ protein genes, including Zo-1, Occludin, Claudin-1 and E-cadherin, were upregulated after co-culture with the gut commensals. For example, *B. thetaiotaomicron* upregulated the mRNA level of Zo-1, Claudin-1 and E-cadherin while *P. distasonis* significantly increased the mRNA level of Occludin. This indicates the regulation of TJ proteins is bacterial species-specific. In addition, commensal species *P. johnsonii* and *A. muciniphila* did not affect mRNA expression of all TJ protein genes test, although it increases the TEER value of cell monolayer. TEER of the cell monolayers cultured on Transwell inserts reflects the tight junctions formed between the epithelail cells. However, TJ proteins belong to a big family and contain many isoforms (33). Therefore, we speculated the TEER increase by *P. johnsonii* and *A. muciniphila* maybe duo to the regulation of TJ proteins other than the tested in this study.

For some probiotics, the realization of their beneficial effects relies upon prior disruption of TJ homeostasis. For example, *E. coli* Nissle 1917 (EcN) did not cause a significant change to the intestinal epithelial barrier function of T84 human intestinal epithelial cells after co-incubation (34). However, EcN supplementation restored the barrier permeability disrupted by enteropathogenic *E. coli* (EPEC). In addition, a commercial probiotic formulation called VSL#3 demonstrated its beneficial effects in a DSS- induced mouse colitis mode (35). Therefore, IL-1β, a pro-inflammatory cytokine, was introduced in this study to simulate an inflamed cell monolayer with a compromised barrier function. IL-1β has been demonstrated with the ability to cause an increase in intestinal epithelial TJ permeability at a physiological concentration (10 ng/mL) (21). Indeed, the addition of IL-1β to the cell monolayer caused a significant drop of TEER value. Furthermore, a decrease in the mRNA expression of TJ protein Occludin and E-cadherin and an increase in Claudin-2 were observed. However, the supplementation of mucin-degrading gut commensals (*P. distasonis*, *P. johnsonii*, *B. salyersiae*, *B. thetaiotaomicron* and *A. muciniphila*) entirely or partially attenuated the IL-1β induced decrease in TEER value. *A. muciniphila* is a known mucin-degrading bacterium and many publications demonstred that this bacterium has a positive effect on intestinal barrier integrity (36). Consistently, *A. muniniphila* here partially restored the IL-1β induced TEER drop and disrupted TJ proteins Occludin and E-cadherin. Notably, we found *B. thetaiotaomicron* and *P. distasonis* test here exert similar protective effect against the stimulation of IL-1β. Particularly, supplementation of *P. distasonis* completely prevented the IL-1β induced TEER drop and disruption of TJ proteins. These observations indicated that increased Occludin, E-cadherin and decreased Claudin-2 expression may contribute to the ability of the gut commensal species to strengthen the epithelial barrier. The genus *Bacteroides* contains the most predominant species of Bacteroidetes order in the human intestinal tract. The health-promoting properties of the species within this genus have been recognized relatively recently with *B. fragilis* being the best studied representative (37). However, results here demonstrate for the first time the barrier-enhancing potential for other *Bacteroides* and *Parabacteroides* spp.

Previous studies demonstrated that IL-1β induced disruption of intestinal TJ barrier function is mediated by the activation of several signaling pathways, such as NF-KB pathway (21). Consistently, the inhibition of IL-1β activated NF-KB pathway by *P. distasonis* and *A. muciniphila* was also observed in this study. Thus, inhibition of the NF-KB activation, resulting in TEER enhancement and restoration of TJ proteins maybe a mechanism mediated the barrier-enhancing potential for the gut commensals in this study. However, *B. thetaiotaomicron* showed no inhibitory effect on IL-1β activated NF-KB pathway, suggesting other signaling pathways involved in regulating TJ proteins.

In contrast to the beneficial effects, two *Bacteroide* species (*B. intestinihominis* and *B. ovatus*) had no significant effects on the TEER value of Caco-2. Notably, one isolate *B. dentium*, together with control strains *L. rhamnosus* ATCC 53103 and pathogenic *E. coli* NCTC 9001, significantly decreased the TEER value after co-culture. Although the exact mechanism by which the isolate *B. dentium* and *L. rhamnosus* ATCC 53103 decrease the TEER is not investigated in this study, the color of cell culture media (DMEM) was observed to become yellow after co-culture, suggesting an acidified environment for the cell monolayers. This result also demonstrated that caution must be taken when interpreting data from *in vitro* models.

In addition, the potential pathogenicity of *Bacteroides* spp. cannot be ignored as the health-promoting characteristics of *Bacteroides* are strictly strain-dependent (38). For example, some enterotoxigenic *B. fragilis* can cause disease such as bacteremia when they have escaped their normal habitats (39). Therefore, further studies are needed to well define the mechanism by which epithelail barrier function is enhanced and the potential risk factors.

In summary, we found the gut commensals with mucin-degrading ability were widely distributed in the human gut microbiota. These gut commensals were tolerated by the epithelial cells and incited low or little level of pro-inflammatory immune response. In contrast, they suppress the inflammatory response induced by *E. coli* through downregulation of NF-KB pathway. Moreover, a total of 7 out of the 10 test gut commensals enhanced the epithelial TJ barrier function, as showed by increased TEER value and upregulated mRNA expression of TJ protein genes. Notably, *P. distasonis* completely protected the epithelial barrier from the challenge of IL-1β by restoring the expression and distribution TJ proteins, while *A. muciniphila* and *B. thetaiotaomicron* could partially restore the disrupted epithelial TJ barrier.

Our findings here provide evidence for the anti-inflammatory and epithelial barrier-enhancing property of mucin-degrading gut commensals, especially *Bacteroides* spp. and *Parabacterodies* spp. These findings enable human gut commensals, the unconventional probiotics with specific phenotype and function, to be promising and new therapeutics in the future for inflammatory bowel disease and other inflammatory disorders.

## Data availability statement

The datasets presented in this study can be found in online repositories. The names of the repository/repositories and accession number(s) can be found below: https://www.ncbi.nlm.nih.gov/, PRJNA635743.

## Ethics statement

The studies involving human participants were reviewed and approved by Joint Chinese University of Hong Kong-New Territories East Cluster Clinical Research Ethics Committee (reference number 2016.707). The patients/participants provided their written informed consent to participate in this study.

## Author contributions

Conceptualization, MI; performance of experiment, MP and NB; data analysis, MP; writing-original draft preparation, MP; writing-review and editing, MP, NB, and MI; supervision, MI; funding acquisition, MI. All authors have read and agreed to the published version of the manuscript.

## Funding

The study was partially supported by a seed fund for gut microbiota research provided by the Faculty of Medicine, The Chinese University of Hong Kong. The funding bodies did not involve in the design of the study and collection, analysis, and interpretation of data and in writing the manuscript.

## Conflict of interest

The authors declare that the research was conducted in the absence of any commercial or financial relationships that could be construed as a potential conflict of interest.

## Publisher’s note

All claims expressed in this article are solely those of the authors and do not necessarily represent those of their affiliated organizations, or those of the publisher, the editors and the reviewers. Any product that may be evaluated in this article, or claim that may be made by its manufacturer, is not guaranteed or endorsed by the publisher.
