# Supplementary material for: Mucin-degrading gut commensals isolated from healthy faecal donor suppress intestinal epithelial inflammation and regulate tight junction barrier function
Source: Front Immunol. 2022 Oct 12;13:1021094. doi: 10.3389/fimmu.2022.1021094 (PMC9597641; doi:10.3389/fimmu.2022.1021094)
Supplement: Supplementary file 1 [file DataSheet_1.docx]

**Supplementary Figures and tables**


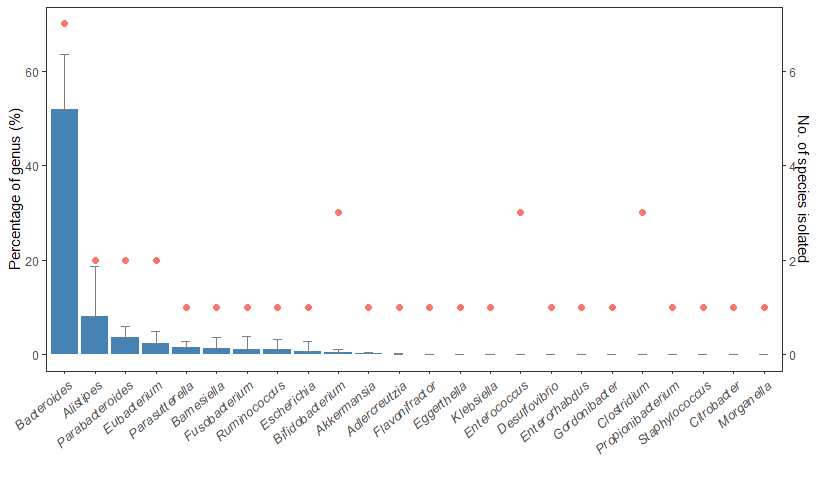


Figure S1 Relative abundance of the 38 bacterial species at the genus level.

Abundance was determined by 16s rRNA amplicon sequencing and based on average relative abundance across faecal samples (n = 27) from three donors. These 38 bacterial species represent 70.35 % of the average relative abundance at the genus level across the faecal samples.


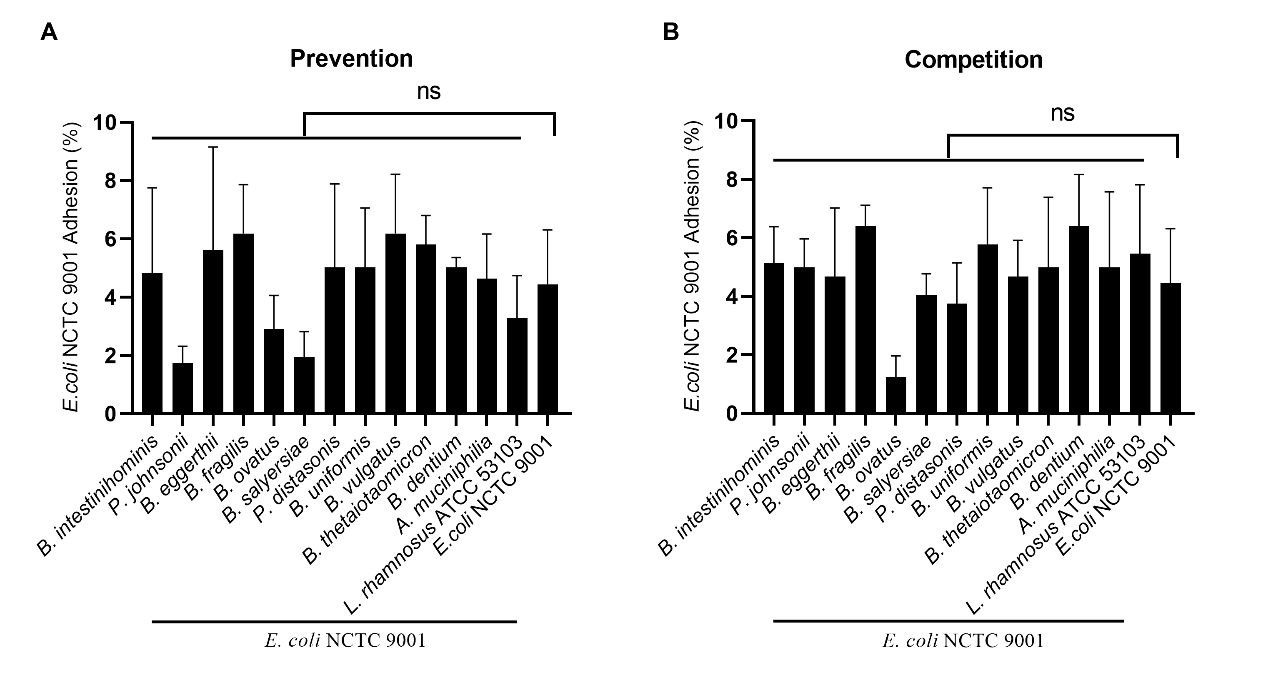


Figure S2 Adhesion of *Escherichia coli* NCTC to HT‐29 cells in the presence of gut commensals under prevention and competition culture conditions.

ns: non-significant compared to the control group as determined by one-way ANOVA followed by Dunnett's test for multiple comparisons.


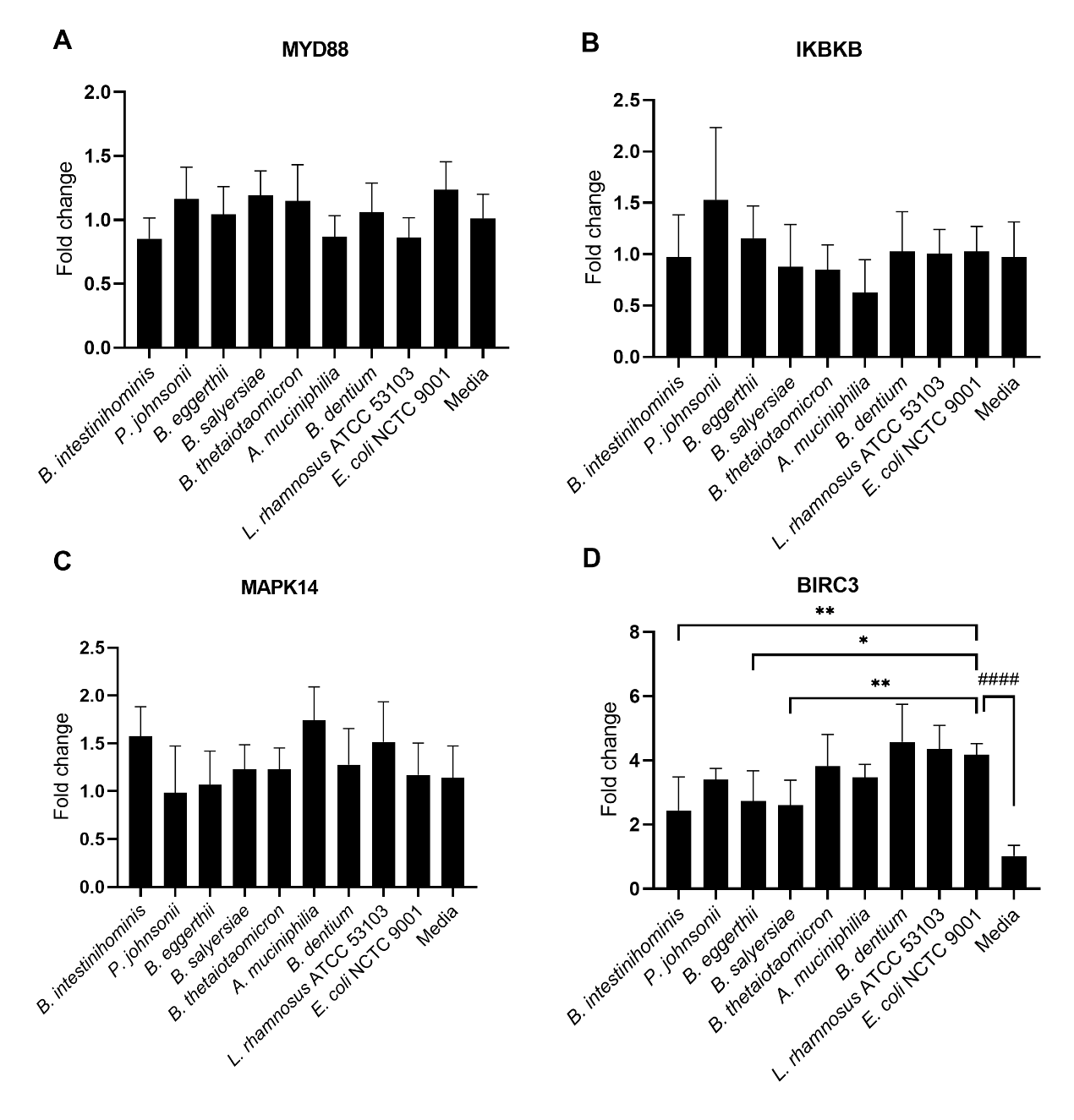


Figure S3 Effect of gut commensals on the mRNA expression of genes involved in NF-κB, MAPK and apoptosis pathway in HT-29 cells that were challenged with pathogenic *E. coli* NCT9001. All data are presented as the mean±SD of three biological replicates with two technical replicates. # p< 0.05, #### p < 0.0001 compared to the control group; * p <0.05, ** p < 0.01, *** p< 0.001, **** p < 0.0001 compared to the *E. coli* NCTC9001 group as determined by one-way ANOVA followed by Dunnett's test for multiple comparisons.


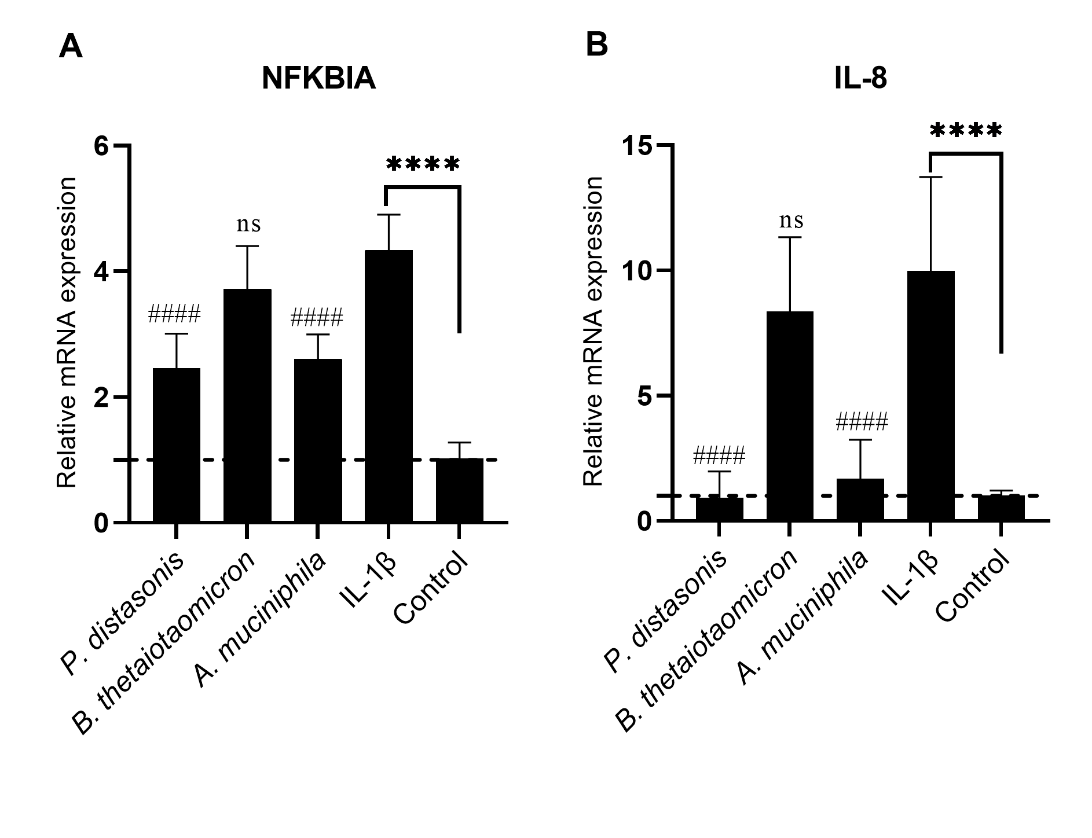


Figure S4 Effect of co-culture with *P. distasonis*, *B. thetaiotaomicron* and *A. muciniphila* on immune response in Caco-2. *P. distasonis* and *A. muciniphila* but not *B. thetaiotaomicron* significantly inhibited the IL-1β induced increase in mRNA expression of NFKBIA (A) and IL-8A (B). **** *p* <0.0001 IL-1β vs. control. #### *p* <0.0001 IL-1β vs. treated groups. Error bars represent the standard deviation of the mean values from at least three replicates. Significance was determined by one-way ANOVA.

**Table S1 Primes for tight junction protein genes**

| **Gene** | **Direction** | **Primer sequence (5′-3′)** | **Tm (ºC)** | **Product length (bp)** | **Accession number** |
| --- | --- | --- | --- | --- | --- |
| Occludin | Forward | AAGAGTTGACAGTCCCATGGCATAC | 63 | 133 | NM_002538 |
|  | Reverse | ATCCACAGGCGAAGTTAATGGAAG | 61 |  |  |
| Cadherin-E | Forward | GCCGAGAGCTACACGTTCA | 60 | 88 | NM_001317185 |
|  | Reverse | GACCGGTGCAATCTTCAAA | 56 |  |  |
| Claudin-1 | Forward | TCACTCCCAGGAGGATGC | 58 | 99 | NM_021101 |
|  | Reverse | GGCAGATCCAGTGCAAAGTC | 59 |  |  |
| Claudin-2 | Forward | GAATCCCGAGCCAAAGACAGAGTG | 63 | 199 | NM_001171095 |
|  | Reverse | TCAGGGAGAACAGGGAAGAAATAA | 59 |  |  |
| Zo-1 | Forward | CAGAGCCTTCTGATCATTCCA | 57 | 69 | NM_001330239 |
|  | Reverse | CATCTCTACTCCGGAGACTGC | 59 |  |  |

**Table S2 Primes for cytokines and immune response.**

| **Gene** | **Direction** | **Primer sequence (5′-3′)** | **Tm (ºC)** | **Product length (bp)** | **Accession number** |
| --- | --- | --- | --- | --- | --- |
| IL-10 | Forward | TGTTGCCTGGTCCTCCTGAC | 62 | 298 | NM000572 |
|  | Reverse | TGGGTCTTGGTTCTCAGCTTG | 60 |  |  |
| IL-8 | Forward | AGACAGCAGAGCACACAAGC | 61 | 62 | NM_00135484 |
|  | Reverse | ATGGTTCCTTCCGGTGGT | 58 |  |  |
| TNF-α | Forward | CTCGAACCCCGAGTGACAAG | 60 | 123 | NM_000594 |
|  | Reverse | TATCTCTCAGCTCCACGCCA | 60 |  |  |
| TGF-β | Forward | GCAGCACGTGGAGCTGTA | 60 | 64 | NM_000660 |
|  | Reverse | CAGCCGGTTGCTGAGGTA | 60 |  |  |
| IKBKB | Forward | AATGAAAGAGCGCCTTGGGA | 60 | 80 | NM_001556 |
|  | Reverse | TGGCAATCTGCTCACCTGTT | 60 |  |  |
| NFKBIA | Forward | AAGTGATCCGCCAGGTGAAG | 60 | 188 | NM_020529 |
|  | Reverse | CTGCTCACAGGCAAGGTGTA | 60 |  |  |
| NFKB1 | Forward | GCTTAGGAGGGAGAGCCCA | 60 | 196 | NM_003998 |
|  | Reverse | GGTATGGGCCATCTGCTGTT | 60 |  |  |
| NFKB2 | Forward | CCTAAGCAGAGAGGCTTCCG | 60 | 133 | NM_002502 |
|  | Reverse | TGGCTGGTCCCTCGTAGTTA | 60 |  |  |
| MYD88 | Forward | GCTCATCGAAAAGAGGTGCC | 60 | 144 | NM_002468 |
|  | Reverse | ACTTGATGGGGATCAGTCGC | 60 |  |  |
| MAPK14 | Forward | TCATTAACAGGATGCCAAGCCA | 60 | 189 | NM_001315 |
|  | Reverse | CAAAGTAGGCATGTGCAAGGG | 60 |  |  |
| JUN | Forward | TGAGTGACCGCGACTTTTCA | 60 | 155 | NM_002228 |
|  | Reverse | TTTCTCTAAGAGCGCACGCA | 60 |  |  |
| CARD9 | Forward | ACACCCAGCTCTCAGACAAAG | 60 | 200 | NM_052813 |
|  | Reverse | CTGCATCTTCCTGAGGGCG | 60 |  |  |
| MAPK14 | Forward | TCATTAACAGGATGCCAAGCCA | 60 | 189 | NM_001315 |
|  | Reverse | CAAAGTAGGCATGTGCAAGGG | 60 |  |  |
| BIRC3 | Forward | TCGCTTGAAAAGACTGGGCT | 60 | 176 | NM_001165 |
|  | Reverse | CCCGAGATTAGACTAAGTCCCTT | 59 |  |  |
| 18S | Forward | TGGCTCATTAAATCAGTTATG | 50 | 88 | M10098 |
|  | Reverse | CGGCATGTATTAGCTCTA | 51 |  |  |
